# SVTRv2X: Enhanced scene text recognition via self-distilled mixture-of-experts

**DOI:** 10.1371/journal.pone.0349085

**Published:** 2026-06-01

**Authors:** Jian Guo, Hanxin Cui, Wengang Tang, Xuehai Zhou, Xing Xu, Qianqian Cheng

**Affiliations:** 1 GUANGXI BEIBU GULF BANK CO., LTD., Nanning, Guangxi, China; 2 Administrative Committee of Nanning New & High-Tech Industrial Development Zone, Nanning, Guangxi, China; University of Sargodha, PAKISTAN

## Abstract

Scene Text Recognition (STR) is a fundamental component of intelligent perception systems and plays a crucial role in a wide range of real-world applications such as autonomous driving, document understanding, and human–computer interaction. STR still faces several challenges in practical applications, including high sensitivity to spatial perturbations, limited representational capacity of lightweight Connectionist Temporal Classification(CTC)-based models, and the difficulty of handling diverse text styles within a single unified architecture. Although SVTRv2 enhances the recognition ability of CTC models through a combination of local and global mixing mechanisms, its robustness and generalization capability remain insufficient when dealing with geometric distortions, complex backgrounds, or text with large stylistic variations. To address these issues, we propose SVTRv2X, an enhanced STR framework built upon SVTRv2 that integrates three complementary improvement modules. The Jumble Module strategically rearranges input patches before the patch embedding stage, fundamentally reducing the model’s reliance on fixed spatial structures and significantly improving robustness to rotated, misaligned, and irregular text. The Self-Distillation Module transfers deep-layer knowledge to shallow features, effectively strengthening early-stage representations while maintaining lightweight inference. The Mixture-of-Experts (MoE) Module expands model capacity through sparsely activated expert networks, allowing specialized processing of different text styles without introducing substantial computational overhead. Extensive experiments demonstrate that SVTRv2X achieves state-of-the-art performance on multiple STR benchmarks, substantially advancing the model’s recognition capability in real-world scene text scenarios.

## 1. Introduction

Scene text recognition, also known as optical character recognition (OCR), plays a crucial role in computer vision due to its wide application in various fields, drawing considerable attention from researchers. Scene text recognition has important applications across various domains. In automated document processing, it improves efficiency by extracting and digitizing text [1], and in assistive tools for visually impaired users, it enables independent access to written information by converting printed or handwritten text into speech or Braille [2]. Additionally, it plays a key role in intelligent transportation systems for license plate and traffic sign recognition [1], and contributes to autonomous driving, human-computer interaction, vehicle plate recognition, and translation services [2].

**Fig 1:**
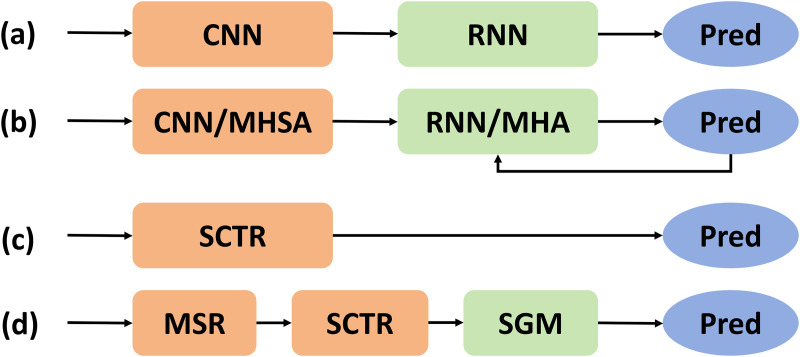
(a) CNN-RNN based models. **(b)** Encoder-Decoder models. MHSA and MHA denote multi-head self-attention and multi-head attention, respectively. **(c)** SVTR. **(d)** SVTRv2.

Deep learning has greatly advanced scene text recognition, significantly improving the accuracy of recognizing different fonts and languages. One category consists of CTC-based models (Fig 1(a)), which offer fast inference but limited robustness [3,4], while the other category comprises attention-based Encoder-Decoder models (Fig 1(b)), which achieve high accuracy at the cost of greater computational overhead. SVTR [5] (Fig 1(c)) introduces a three-stage backbone network that employs local and global mixing operations to capture stroke-level features and long-range dependencies, enabling efficient linear prediction. SVTRv2 [6] (Fig 1(d)) further enhances performance with Multi-Scale Resizing (MSR) and a Feature Rearrangement Module (FRM), strengthening CTC-based recognition while maintaining a simple structure and efficient inference.

Recent works [7–9] on scene understanding and non-Latin text recognition have achieved remarkable results. Despite the rapid development of scene text recognition (STR) models, several practical challenges remain unresolved. Current methods are highly sensitive to spatial perturbations, limiting their robustness when text appears in rotated, misaligned, or irregular layouts. Lightweight CTC-based models, while efficient, often suffer from insufficient representational capacity, making it difficult to capture complex character patterns. Moreover, handling diverse text styles within a single unified architecture remains challenging, as existing models struggle to generalize across geometric distortions, complex backgrounds, or large stylistic variations. Even advanced architectures like SVTRv2, which leverage local and global mixing mechanisms to enhance recognition, still exhibit noticeable limitations in robustness and generalization under real-world conditions.

To address these issues, We present SVTRv2X, an enhanced framework built upon SVTRv2 that integrates three synergistic, modules to boost model performance. First, we introduce a Jumble Module before the patch embedding stage to strategically augment the input data, thereby bolstering the model’s robustness against spatial perturbations. Second, we incorporate a Self-Distillation Module to facilitate knowledge transfer from deeper to shallower layers, which enriches the model’s feature representation capabilities. Finally, a Mixture of Experts (MoE) module is employed to enable specialized processing of textual features from diverse scenes, achieving a “divide and conquer” recognition strategy. Experimental results demonstrate that SVTRv2X significantly improves recognition accuracy on benchmark datasets compared to SVTRv2, all while maintaining its highly competitive inference speed.

Our contributions are summarized as follows:

1. We propose a Jumble Module placed before the patch embedding stage, which strategically rearranges local image patches during training. This encourages the model to learn position-invariant and robust features, significantly improving resilience to rotated, misaligned, and irregular text.
2. We integrate a Self-Distillation Module that transfers knowledge from deeper layers to shallower ones during training, enhancing early-stage feature representations while maintaining a lightweight architecture and efficient inference.
3. We employ a Mixture-of-Experts (MoE) Module, where a sparsely activated set of expert subnetworks dynamically processes each input. This enables specialized modeling of diverse text styles without introducing substantial computational overhead.
4. Extensive experiments on multiple open-source STR benchmarks demonstrate that SVTRv2X achieves state-of-the-art (SOTA) performance, improving recognition accuracy while maintaining highly efficient inference.

## 2. Related work

### 2.1 Scene text recognition

Scene text recognition has drawn huge attention of many researchers in computer vision field and has been studied for many decades. Traditionally, as before deep learning era, scene text recognition can be divided into two parts, text detection and text recognition. For text detection, Chen et al. [5] use texture based method, which use texture features of text to detect text region in the image. Lee et al. [6] use Sliding Window, which use different size of windows sliding over image, classifying each window containing text or not. For text recognition, tradition methods use character features for classification, such as character key-points (Phan et al.) [10] and bottom-up and top-down cues (Mishra et al.) [11]. Compared with deep learning methods, these method are at disadvantage in accuracy.

After deep neural networks’ rapid and tremendous development and application, performance of scene text recognition greatly improves. Huang et al. [12] use convolutional neural networks (CNNs) to detect text in images. Zhan et al. [13] and Liao et al. [14] use methods and techniques inspired by object detection to achieve scene text recognition. He et al. [15] use RNN-based models for text recognition. Lee et al. [16] proposed a Encoder-Decoder model, using recursive convolutional layers as encoder and RNN as decoder. Aberdam et al. [17] propose CLIP Text Recognition using vision-language transformer model to provide rich scene-level information to the crop-based recognizer in scene text recognition, thus obtain entire image informantion in case of poor-quality text. Xue et al. [18] bring up I2C2W, decomposing the task into image-to-character and character-to-word which detect characters in an non-sequential way, with performance well in sense with noises. Yu et al. [19] propose an architecture consisting of a CCR-CLIP(Chinese character recognition) pre-training stage and a CTR(Chinese Text Recognition) stage specialized for scene Chinese text.

To address the recognition challenges posed by the diversity of text instances, numerous recent works have enhanced CTC-based methods. One prevailing strategy involves introducing rectification modules, inspired by prior work. These modules aim to transform irregular text into a more regular, recognizable format through geometric or perspective corrections. Another widely adopted approach is to integrate an attention-based decoder. Such decoders leverage attention mechanisms to sequentially decode visual features, allowing them to dynamically attend to and identify each character, thus offering a more flexible alignment mechanism than conventional CTC. However, current works still have weakness and unsolved problems.

### 2.2 Patch-level spatial augmentation

Data augmentation plays a crucial role in enhancing the generalization ability and robustness of vision models. Existing techniques span from global image-level transformations to fine-grained patch manipulation and mixing-based sample synthesis. This section reviews two representative lines of work—traditional augmentation methods and mixup-based augmentation—with emphasis on how they manipulate global or local patches to improve model robustness and representation diversity.

Traditional data augmentation typically targets individual images, performing basic geometric transformations and color conversions [20]. Image-level methods such as random flipping and random cropping are among the most widely used augmentations and consistently improve the generalization performance of neural networks on clean data. Other appearance-based techniques—including adjustments of sharpness, brightness, and Gaussian noise—have also been utilized in various works [21]. With the increasing interest in combining multiple augmentation strategies, automatic augmentation frameworks such as AutoAugment [22], RandAugment [23], and TrivialAugment [24] have been proposed to search or generate diverse combinations of augmentations. Beyond global transformations, patch-level augmentation focuses on perturbing local spatial structures. Cutout [25] randomly masks a portion of the image, Patch Gaussian [26] injects Gaussian noise into a selected patch to balance accuracy and robustness, while Random Erasing [27] introduces varying levels of occlusion without parameter learning. Although effective, these traditional augmentation methods generally preserve the overall structure of the image and thus provide limited diversification of feature representations.

Another important direction is mixup-based augmentation, which synthesizes new samples by combining multiple images or patches. Linear mixing, represented by Mixup [28], linearly interpolates two images and their labels in a random ratio, acting as a powerful regularizer that improves model accuracy. Manifold Mixup [29] extends this idea to hidden-layer features to preserve the manifold structure among samples, while Subgroup Mixup [30] performs pairwise mixup to encourage fair and accurate decision boundaries across different subgroups. In terms of nonlinear mixing, CutMix [31] replaces a randomly cropped patch of one image with a patch from another image, with labels mixed proportionally to patch areas. PuzzleMix [32] and SaliencyMix [33] incorporate saliency maps to guide patch selection, ensuring that discriminative regions remain intact. AutoMix [34] further generates mixed samples adaptively based on learned feature maps and mixing ratios in an end-to-end manner. Despite producing rich and reliable sample variations, mixup-based methods typically incur non-negligible computational overhead and introduce challenges in label assignment due to the involvement of multiple images.

## 3. Materials and methods

### 3.1 SVTRv2

**Fig 2:**
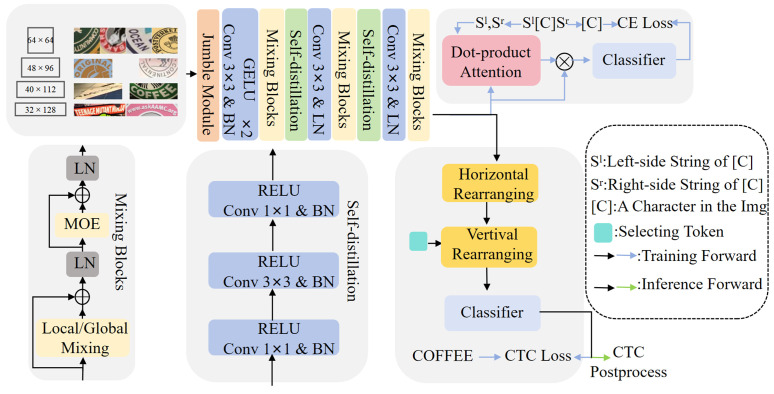
The architecture of the SVTRv2X. Reprinted from [35] under the MIT License, original copyright © 2021 FudanVIC Team.

This chapter delves into the technical details of the enhanced SVTRv2X architecture. Fig 2 shows the overall of our proposed SVTRv2X architecture. We begin with an overview of the baseline SVTRv2 model and then introduce the two main contributions of this work: the jumble module and the self-distillation module. Fig 2 illustrates the overall architecture of our proposed SVTRv2X. SVTRv2X evolves from the highly efficient SVTRv2 model. The visual backbone of SVTRv2 is composed of three stages, with each stage containing six Mixing Blocks designed to progressively extract hierarchical visual features. To extract discriminative representations, SVTRv2 meticulously designs two types of mixing blocks: Local Mixing and Global Mixing. Notably, to accommodate the processing of multi-sized text instances, SVTRv2 innovatively replaces computationally intensive windowed attention [14] with two consecutive group convolutions for its local mixing operation. This design efficiently models positional information and effectively captures local character features such as edges, textures, and strokes. Building upon this strong baseline, we integrate three enhancement modules to construct the SVTRv2X framework.

### 3.2 Jumble Module

We introduce a patch-level shuffling module before the patch embedding layer, which is applied exclusively during the training phase. Unlike conventional data augmentation strategies that rely on cross-image mixing, the proposed module operates within each individual image and performs controlled spatial perturbations to enhance representation robustness.

Given an input image $I\in {\mathbb{R}}^{H\times W\times C}$, it is first divided into *N* non-overlapping patches of size *p* × *p*:


$$\begin{array}{c} N=\frac{H}{p}\times \frac{W}{p}. \end{array}$$ (1)

Let $\{P_{i}{\}}_{i=1}^{N}$ denote the set of patches. Instead of globally shuffling all patches, we randomly select a subset 𝒮⊂{1,…,N} with a predefined ratio P∈[0,1], In our experiments, the shuffle ratio *P* is set to 0.1 by default, and only permute the patches within this subset:


$$\begin{array}{c} {\tilde{P}}_{i}=\left\{ \begin{array}{cc} P_{\pi (i)}, & \text{if }i\in 𝒮,\\ P_{i}, & \text{otherwise,} \end{array}\right. \end{array}$$ (2)

where π is a random permutation defined over 𝒮. The resulting ${\tilde{P}}_{i}$ form the augmented image $\tilde{I}$.

This design introduces a partial patch shuffling strategy, where only a portion of patches are rearranged while the remaining patches preserve their original spatial positions. Such a mechanism maintains a balance between structural consistency and spatial disruption. Importantly, each patch remains intact, meaning that local visual patterns are preserved while inter-patch spatial relationships are selectively perturbed.

By disrupting local spatial continuity in a controlled manner, the model is encouraged to rely less on fixed spatial adjacency and instead learn more robust and position-invariant representations. This is particularly beneficial for scene text recognition, where text may appear with irregular layouts, distortions, or perspective variations.

**Fig 3:**
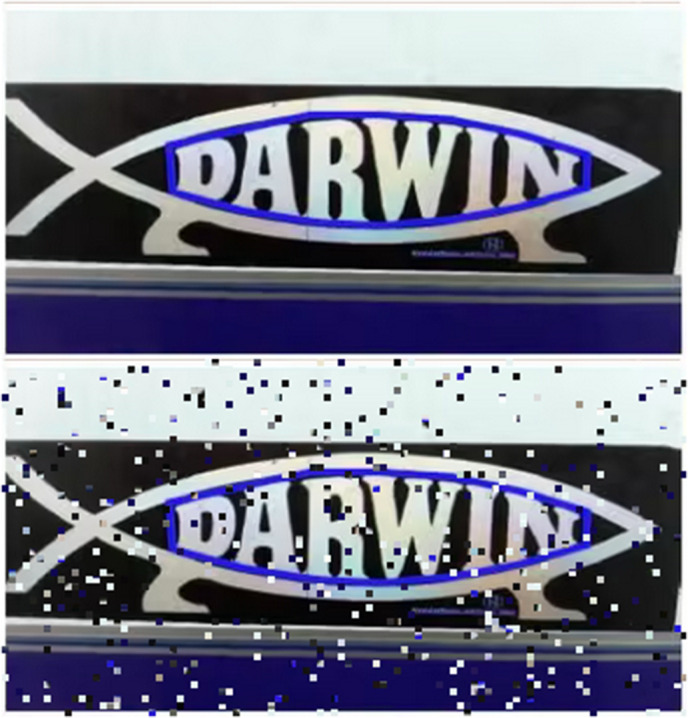
Illustration of the Jumble Module. The input image is transformed into a partially shuffled version.

In practice, JM is only activated during training and introduces negligible computational overhead. During inference, the input is processed without any modification, ensuring full compatibility with standard patch embedding pipelines. Fig 3 illustrates examples of augmented images generated by the proposed module.

The Jumble Module can be viewed as a lightweight structural regularization technique. By exposing the model to diverse spatial configurations within each image, it effectively improves generalization ability and alleviates overfitting, without introducing label inconsistency or cross-image artifacts.

### 3.3 Self-distillation module (SDM)

Some recent works on knowledge distillation and multi-scale attention mechanisms [36,37] have inspired the design of our Self-distillation module. To facilitate feature learning, we introduce a self-distillation module (SDM) integrated into the baseline SVTRv2 architecture after stage 1 and stage 2, following the design paradigm of SVTRv2. Let *F*_1_ and *F*_2_ denote the intermediate features extracted from stage 1 and stage 2, respectively, where $F_{1}\in {\mathbb{R}}^{H_{1}\times W_{1}\times D_{1}}$ and $F_{2}\in {\mathbb{R}}^{H_{2}\times W_{2}\times D_{2}}$.

To enable effective feature alignment, both features are first projected into a shared latent space via a 1 × 1 convolution:


$$\begin{array}{c} {\hat{F}}_{k}={\text{Conv}}_{1\times 1}(F_{k}),\;k=1,2 \end{array}$$ (3)

where ${\hat{F}}_{k}\in {\mathbb{R}}^{H^{\prime }\times W^{\prime }\times D^{\prime }}$. A subsequent non-overlapping 3 × 3 convolution is applied to enhance local spatial interactions within each feature map:


$$\begin{array}{c} {\tilde{F}}_{k}={\text{Conv}}_{3\times 3}^{\text{non-overlap}}({\hat{F}}_{k}) \end{array}$$ (4)

Finally, another 1 × 1 convolution is used to match the feature dimensions:


$$\begin{array}{c} F_{k}^{\prime }={\text{Conv}}_{1\times 1}({\tilde{F}}_{k}) \end{array}$$ (5)

Unlike traditional distillation methods that rely on an external teacher model, our SDM adopts an internal self-distillation strategy, where deeper features serve as implicit teachers for shallower ones. Specifically, we use the transformed feature $F_{2}^{\prime }$ as the teacher to supervise $F_{1}^{\prime }$. The self-distillation loss is defined as:


$$\begin{array}{c} L_{\text{sd}}=\|F_{1}^{\prime }-\text{sg}(F_{2}^{\prime }){\|}_{2}^{2} \end{array}$$ (6)

where sg(·) denotes the stop-gradient operation. In this formulation, the number of supervised feature levels is fixed as *N* = 2, corresponding to stage 1 and stage 2.

To stabilize training, we adopt a two-phase training strategy. In the first phase, the model is trained without the self-distillation loss to learn reliable feature representations. In the second phase, we enable *L*_sd_ and fine-tune the model for an additional 20 epochs. This design is motivated by the observation that applying self-distillation at early stages may introduce noisy supervision due to immature feature representations. Empirically, this two-stage strategy leads to more stable convergence and improved performance compared to joint training from scratch, as demonstrated in our ablation study.

### 3.4 Mixture of experts module

**Fig 4:**
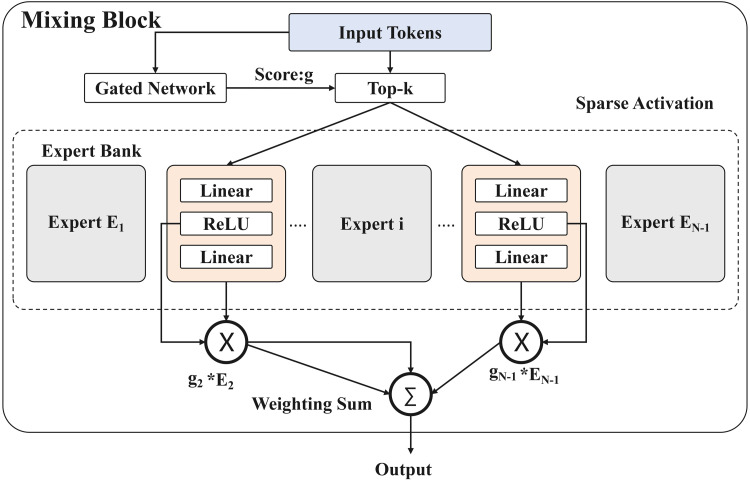
MOE structure.

Within the Mixing blocks, we replace the MLP with a Sparse Mixture-of-Experts (MoE) layer, as illustrated in Fig 4. The MoE layer consists of a lightweight gating network and *N* parallel expert sub-networks. Unlike a standard FFN that applies the same transformation to all input tokens, the MoE layer dynamically routes each token to a small subset of the experts. This sparse activation mechanism significantly increases the model capacity while keeping the computational cost nearly unchanged.

Formally, given an input token representation $x\in {\mathbb{R}}^{D}$, the gating network computes a score for each expert:


$$\begin{array}{c} g=\text{softmax}(W_{g}x+b_{g})\in {\mathbb{R}}^{N}, \end{array}$$ (7)

where *W*_*g*_ and *b*_*g*_ denote the gating parameters. To enforce sparsity, only the top-*K* experts (typically *K* = 1 or 2) are selected for each token. Let 𝒯(g) denote the indices of the top-*K* values in *g*. The routed output is then computed as:


$$\begin{array}{c} y=\sum_{i\in 𝒯(g)}g_{i}\;E_{i}(x), \end{array}$$ (8)

where $E_{i}(\cdot )$ represents the *i*-th expert network, typically a two-layer feed-forward module:


$$\begin{array}{c} E_{i}(x)=W_{2,i}\;\sigma (W_{1,i}x+b_{1,i})+b_{2,i}. \end{array}$$ (9)

In our experiments, the MoE module uses 4 experts. We adopt a top-1 routing strategy for activation sparsity. A load balancing loss is applied during training to encourage uniform expert utilization, but it is not applied during inference.

By activating only a few experts per token, the MoE layer adopts a “divide-and-conquer” strategy that allows distinct experts to specialize in different textual patterns such as font variations, stroke styles, distortions, or noise types. This specialization not only improves the expressive power of the Transformer blocks but also significantly enhances recognition accuracy with only marginal additional computational cost (FLOPs).

### 3.5 Loss function

During training, the SVTRv2X model is optimized by minimizing a combined loss function that integrates multiple supervision signals, including the CTC loss, semantic guidance module (SGM) loss, and self-distillation loss. The Connectionist Temporal Classification (CTC) loss [38] is applied to handle the misalignment between the input feature sequence and the target character sequence. Given the input feature sequence $X\in {\mathbb{R}}^{T\times D}$ and the target label sequence *Y*, the CTC loss is defined as:


$$\begin{array}{c} L_{\text{ctc}}=-\log\sum_{\pi \in {ℬ}^{-1}(Y)}P(\pi |X) \end{array}$$ (10)

where π represents a valid alignment path, and ${ℬ}^{-1}(Y)$ denotes the set of all paths that collapse to the target sequence *Y* after removing repeated characters and blank tokens.

To incorporate linguistic context during training, the semantic guidance module applies cross-entropy supervision to both left-to-right and right-to-left predictions of the character sequence. The SGM loss is formulated as:


$$\begin{array}{c} L_{\text{sgm}}=\frac{1}{2L}\sum_{i=1}^{L}\left(\text{CE}({\tilde{Y}}_{i}^{l},c_{i})+\text{CE}({\tilde{Y}}_{i}^{r},c_{i})\right) \end{array}$$ (11)

where CE(·) denotes the cross-entropy loss, *L* is the sequence length, ${\tilde{Y}}_{i}^{l}$ and ${\tilde{Y}}_{i}^{r}$ are the left-to-right and right-to-left predictions, and *c*_*i*_ is the ground truth character at position *i*.

To further enhance feature representation, the self-distillation module encourages knowledge transfer across different stages. Specifically, deeper features are used to guide shallower ones. Let $F_{1}^{\prime }$ and $F_{2}^{\prime }$ denote the transformed features from stage 1 and stage 2, respectively. The self-distillation loss is defined as:


$$\begin{array}{c} L_{\text{sd}}=\|F_{1}^{\prime }-\text{sg}(F_{2}^{\prime }){\|}_{2}^{2} \end{array}$$ (12)

where sg(·) denotes the stop-gradient operation. In this work, the number of supervised feature levels is fixed as *N* = 2, corresponding to stage 1 and stage 2.

To ensure balanced expert utilization and avoid expert collapse, we follow standard practices and introduce a load-balancing auxiliary loss:


$$\begin{array}{c} L_{\text{load}}=N\sum_{i=1}^{N}f_{i}\;p_{i}, \end{array}$$ (13)

where *f*_*i*_ is the fraction of tokens assigned to expert *i*, and *p*_*i*_ is the average gate probability for that expert.

The total training loss combines these components with weighting factors $\lambda_{1}$, $\lambda_{2}$, and $\lambda_{3}$, formulated as:


$$\begin{array}{c} L=\lambda_{1}L_{\text{ctc}}+\lambda_{2}L_{\text{sgm}}+\lambda_{3}L_{\text{sd}} \end{array}$$ (14)

In our experiments, $\lambda_{1}$, $\lambda_{2}$, and $\lambda_{3}$ are set to 0.1, 1.0, and 0.5, respectively, which balances the contributions of alignment supervision, linguistic guidance, and self-distillation.

## 4. Experiments

To evaluate the effectiveness of the enhanced SVTRv2X, we conducted scene text recognition experiments on a baseline dataset. We compared our proposed approach to state-of-the-art models, including baseline SVTR and other lightweight architectures.

### 4.1 Experimental setup

#### 4.1.1 Dataset.

We evaluated our approach on the IIIT 5K-Word Dataset [39], ICDAR 2013 Dataset [40] and the Chinese Text Recognition Scene Dataset [35].

The IIIT 5K-Word Dataset (IIIT5K) is a widely used benchmark for scene text recognition, collected from Google image search. It contains 5,000 cropped word images. The dataset covers a large variety of fonts, backgrounds, distortions, and noise, making it suitable for evaluating robustness in natural scene text recognition.

The ICDAR 2013 Dataset (ICDAR2013) is another standard benchmark introduced in the ICDAR Robust Reading Competition. It contains high-quality word images captured from real-world scenes. Compared to IIIT5K, ICDAR2013 is relatively cleaner and is often used to evaluate recognition performance under less noisy conditions.

**Fig 5:**
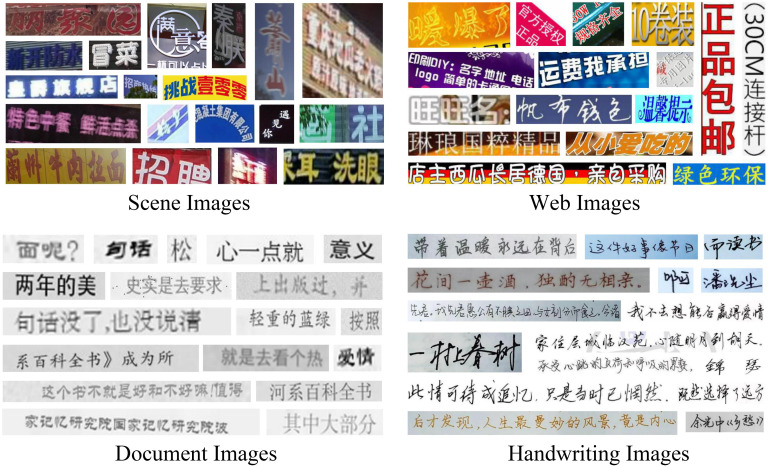
Examples of the Chinese Text Recognition Scene Dataset. Reprinted from [35] under the MIT License, original copyright © 2021 FudanVIC Team.

Fig 5 and Table 1 illustrates the four datasets used in the Chinese Text Recognition Scene Dataset, including Scene, Web, Document, and Handwriting datasets. A unified processing pipeline was applied across all datasets to ensure consistency. Below we provide a concise description of their sources and construction, while detailed statistics are summarized in Table 1.

**Table 1:** Statistics of the Chinese Text Recognition Scene Dataset.

Dataset	Train	Val	Test
Scene	509,164	63,645	63,646
Web	112,471	14,059	14,059
Document	400,000	50,000	50,000
Handwriting	74,603	18,651	23,389

The Scene dataset is constructed by combining several publicly available Chinese scene text benchmarks, including RCTW [41], ReCTS [42], LSVT [43], ArT [44], and CTW [45]. All images were cropped into text-line samples using the official annotations. For RCTW, only the training split was used since the test set does not provide ground-truth labels. For LSVT, we selected only the fully annotated subset. The cropped samples from all five datasets were merged and randomly divided into training, validation, and testing subsets with an 8:1:1 ratio.

The Web dataset is derived from MTWI, which contains web text images spanning 17 categories from Taobao. We cropped text-line samples from the training split and then manually partitioned them into three subsets using an 8:1:1 ratio to account for the dataset’s diverse styles and typography.

The Document dataset consists of synthetic text images generated using the Text Render engine. Text sequences of lengths 1–15 were uniformly sampled, and the corpus was compiled from open-domain sources such as Wikipedia, movie subtitles, Amazon reviews, and encyclopedic entries. The generated images were randomly divided with an 8:1:1 split.

The Handwriting dataset is based on SCUT-HCCDoc, which contains naturally captured handwritten Chinese text. Following common practice, the original training portion was further divided into training and validation subsets with a 4:1 ratio, while the official test set was retained unchanged.

#### 4.1.2 Implementation details.

We implemented our model using the PyTorch framework and trained it with the Adam optimizer. The initial learning rate was set to 0.00065 and decayed using a cosine annealing schedule to ensure stable convergence. All models were trained with a batch size of 8.

All experiments were conducted on a workstation equipped with 64 GB RAM and an NVIDIA RTX3090 GPU. The training environment was built on Ubuntu 20.04, CUDA 12.1, and PyTorch 2.1, ensuring full support for mixed-precision acceleration and efficient large-scale training.

### 4.2 Ablation study

To precisely quantify the contribution of the Jumble Module (JM), we conducted an ablation study by integrating it into the SVTRv2 baseline. As detailed in the Table 2, this addition results in a tangible improvement, elevating the average accuracy from 79.45% to 79.72%. A more granular analysis of the per-category performance reveals the module’s core strength: enhancing spatial robustness. The most significant gains are concentrated on datasets known for their geometric and stylistic irregularities. Specifically, we observe a 0.5% absolute accuracy improvement on the Sence dataset (from 77.8% to 78.3%) and a 0.4% improvement on the Handwriting dataset (from 62.0% to 62.4%). In contrast, its impact on the highly structured Document dataset is negligible, which is expected. To further assess its contribution within the full framework, we compare the configurations with and without JM under the same setting of other modules. Specifically, adding JM on top of the SDM + MoE configuration improves the average accuracy from 81.03% to 81.64% (+0.61%), with consistent gains observed across all subsets (e.g., Scene: + 1.1%, Web: + 0.6%, Handwriting: + 0.8%). These results indicate that JM serves as a complementary module that enhances robustness by introducing controlled spatial perturbations. While its standalone improvement is relatively smaller compared to SDM, its contribution becomes more pronounced when combined with other modules, leading to a more generalized and stable model.

**Table 2:** Ablation study results on different Text Recognition Scenarios.

Baseline	JM	SDM	MoE	Scene	Web	Document	Handwriting	Avg.
✔				77.8	78.8	99.3	62.0	79.45
✔	✔			78.3	78.9	99.3	62.4	79.72
✔		✔		79.8	79.8	99.4	64.3	80.81
✔			✔	78.8	79.0	99.4	63.0	80.05
✔		✔	✔	80.0	80.6	99.4	64.1	81.03
✔	✔	✔	✔	81.1	81.2	99.4	64.9	81.64

The effectiveness of the Self-distillation Module (SDM) is demonstrated through a significant performance leap across the board. Integrating SDM elevates the average accuracy by a substantial 1.36%, from 79.45% to 80.81%, marking it as a highly impactful component of our framework. This module acts as a powerful, universal feature enhancer, with its benefits being particularly pronounced on the more challenging data categories. The most dramatic improvement is seen on the Handwriting dataset, where accuracy surges by 2.3% (from 62.0% to 64.3%). Similarly, the Sence dataset benefits from a large 2.0% gain (from 77.8% to 79.8%). These results strongly suggest that the internal knowledge transfer from deeper “teacher” layers to shallower “student” layers successfully enriches the feature representations at every stage. This process not only improves final prediction accuracy but also acts as an implicit regularizer, leading to a more generalized and powerful model without incurring any additional inference overhead.

We investigate the impact of replacing the standard dense Feed-Forward Networks (FFNs) with our proposed Mixture of Experts (MoE) layers. The results clearly demonstrate the value of this architectural shift, with the MoE module boosting the average accuracy from 79.45% to 80.05%. The strength of the MoE architecture lies in its ability to increase model capacity efficiently, allowing for specialization. This is reflected in the balanced and significant improvements on the two most diverse and challenging datasets: both Sence and Handwriting accuracy increase by a solid 1.0% each (to 78.8% and 63.0%, respectively). This outcome supports our assertion that the “divide-and-conquer” approach, where different experts learn to handle distinct data patterns (e.g., varied fonts, noises, or styles), provides a significant advantage over a monolithic FFN. The MoE module successfully enhances the model’s expressive power and its ability to generalize to complex text instances, all while maintaining computational efficiency.

### 4.3 Comparison with state-of-the-arts

In this study, we perform extensive comparative experiments with existing methods to validate the effectiveness of the proposed approach. Furthermore, the model size settings are aligned with those of SVTRv2, ensuring fairness and consistency in the experimental comparisons.

According to the performance results summarized in Table 3, the SVTRv2X series achieves leading accuracy across different Chinese text recognition scenarios. The smallest variant, SVTRv2X-T, reaches an average accuracy of 81.64%, already surpassing strong baseline models such as DPTR (80.73%), CDistNet (80.58%), CPPD (78.55%), and IGTR (81.74%). Scaling up to SVTRv2X-S and SVTRv2X-B, the average accuracy further increases to 83.25% and 84.68%, respectively, establishing a new state-of-the-art across the benchmark. These results demonstrate that the proposed enhancement modules—Jumble, MoE, and self-distillation—consistently strengthen feature representations and improve performance across diverse text categories.

**Table 3:** Performance comparison on different Chinese Text Recognition Scenarios (%).

Model	Venue	Encoder	Scene	Web	Document	Handwriting	Avg.
ASTER [46]	TPAMI19	ResNet+LSTM	61.3	51.7	96.2	37.0	61.55
MORAN [47]	PR19	ResNet+LSTM	54.6	31.5	86.1	16.2	47.10
SAR [48]	AAAI19	ResNet+LSTM	59.7	58.0	95.7	36.5	62.48
SEED [49]	CVPR20	ResNet+LSTM	44.7	28.1	91.4	21.0	46.30
MASTER [50]	PR21	CNN	62.8	52.1	84.4	26.9	56.55
ABINet [51]	CVPR21	ResNet + TF3	66.6	63.2	98.2	53.1	70.28
TransOCR [52]	CVPR21	Transformer	71.3	64.8	97.1	53.0	71.55
CCR-CLIP [19]	ICCV23	ResNet+Transformer	71.3	69.2	98.3	60.3	74.78
DCTC [53]	AAAI24	SVTR-L	73.9	68.5	99.4	51.0	73.20
CAM [54]	PR24	ConvNeXtV2	76.0	69.3	98.1	59.2	76.80
LISTER [55]	ICCV23	FocalNet-B	79.4	79.5	99.2	58.0	79.02
DPTR [56]	MM24	ViT	80.0	79.6	98.9	64.4	80.73
CDistNet [57]	IJCV24	ResNet + TF3	80.0	79.5	99.1	63.7	80.58
CPPD [58]	TPAMI25	SVTR-B	78.4	79.3	98.9	57.6	78.55
IGTR [59]	TPAMI25	SVTR-B	82.0	81.7	99.5	63.8	81.74
SVTRv2 [6]	ICCV25	SVTRv2-B	83.5	83.3	99.5	67.0	83.31
SVTRv2X	–	SVTRv2X-T	81.1	81.2	99.4	64.9	81.64
		SVTRv2X-S	83.4	83.5	99.4	66.7	83.25
		SVTRv2X-B	84.9	85.0	99.5	69.3	84.68

In the Scene category, which is widely considered the most challenging due to complex backgrounds, occlusion, illumination variation, and geometric distortions, SVTRv2X-T achieves 81.1% accuracy, already higher than CDistNet (80.0%), CPPD (78.4%), and DPTR (80.0%). Scaling to SVTRv2X-S and SVTRv2X-B, the accuracy increases to 83.4% and 84.9%, respectively. This indicates that the Jumble module effectively enhances robustness against

spatial perturbations, while the MoE module enables specialization for complex visual patterns, collectively boosting generalization in natural scenes.

For Web images, SVTRv2X achieves 81.2%, 83.5%, and 85.0% for the T, S, and B variants, surpassing CDistNet (79.5%), CPPD (79.3%), and IGTR (81.7%). This highlights the importance of the self-distillation module, which improves consistency within the latent feature space and enhances robustness across heterogeneous text styles.

In the Document category, all models perform relatively well due to the clean and structured nature of document text. SVTRv2X-T and SVTRv2X-S achieve 99.4%, while SVTRv2X-B slightly improves to 99.5%, consistently exceeding CPPD (98.9%) and CDistNet (99.1%) and matching the highest scores of IGTR (99.5%). This suggests that the enhancement modules maintain stable feature representations even in less challenging scenarios.

In the Handwriting category, characterized by diverse writing styles and irregular strokes, SVTRv2X-T, S, and B achieve 64.9%, 66.7%, and 69.3%, respectively, outperforming DPTR (64.4%), CDistNet (63.7%), CPPD (57.6%), and IGTR (63.8%). The MoE module’s expert specialization effectively handles stroke-level variability, while the Jumble module contributes spatial robustness, together enhancing the model’s capacity to generalize to irregular handwritten text.

Table 4 presents the performance comparison of our proposed SVTRv2X models against several state-of-the-art STR methods on standard Latin-based datasets, including IIIT5K and ICDAR2013. From the results, it can be observed that the SVTRv2X series consistently achieves competitive or superior performance across different model scales.

**Table 4:** Performance comparison on common dataset (%).

Model	Venue	Encoder	IIIT5k	ICDAR2013	Avg.
ASTER [46]	TPAMI19	ResNet+LSTM	96.1	94.9	95.5
MORAN [47]	PR19	ResNet+LSTM	96.7	94.6	95.7
SAR [48]	AAAI19	ResNet+LSTM	98.1	96.7	97.4
SEED [49]	CVPR20	ResNet+LSTM	96.5	94.2	95.3
ABINet [51]	CVPR21	ResNet + TF3	98.5	97.7	98.1
CAM [54]	PR24	ConvNeXtV2	98.2	96.6	97.4
LISTER [55]	ICCV23	FocalNet-B	98.8	98.6	98.7
CDistNet [57]	IJCV24	ResNet + TF3	98.7	97.8	98.3
CPPD [58]	TPAMI25	SVTR-B	99.0	98.2	98.6
IGTR-AR [59]	TPAMI25	SVTR-B	98.7	98.1	98.4
SVTRv2 [6]	ICCV25	SVTRv2-B	99.2	98.7	99.0
SVTRv2X	–	SVTRv2X-T	99.0	98.2	98.6
		SVTRv2X-S	99.4	98.8	99.1
		SVTRv2X-B	99.6	98.9	99.2

Specifically, SVTRv2X-T achieves 99.0% accuracy on IIIT5K and 98.2% on ICDAR2013, comparable to previous top-performing models such as CPPD and IGTR-AR. Scaling up to SVTRv2X-S, the model reaches 99.4% on IIIT5K and 98.8% on ICDAR2013, surpassing most existing methods. The largest variant, SVTRv2X-B, achieves 99.6% on IIIT5K and 98.9% on ICDAR2013, leading to an average accuracy of 99.2%, which is on par with or slightly better than SVTRv2-B and other recent SOTA approaches such as LISTER and CPPD.

These results demonstrate that our proposed SVTRv2X framework not only maintains strong performance on Chinese STR datasets but also generalizes effectively to Latin-based text recognition, highlighting the robustness and versatility of the model across different languages and scripts.

## 5. Discussion

### 5.1 Visual comparison of Chinese Text Recognition

**Fig 6:**
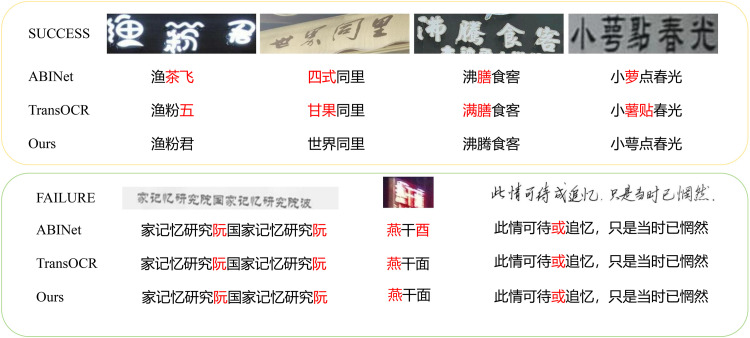
Visual comparison of Chinese Text Recognition: SVTRv2X vs. ABINet and TransOCR on success and failure cases. Reprinted from [35] under the MIT License, original copyright © 2021 FudanVIC Team.

To further demonstrate the effectiveness of SVTRv2X, we compare its recognition results with two representative models, ABINet and TransOCR, on challenging Chinese text samples as shown in the Fig 6. In the successful cases, SVTRv2X consistently recognizes fine-grained characters with similar strokes or radicals, which are often misclassified by ABINet and TransOCR. For example, characters with subtle bifurcated strokes or nested radicals are accurately distinguished by SVTRv2X, whereas ABINet may confuse these characters due to less discriminative feature representations, and TransOCR often struggles under complex backgrounds or non-standard fonts.

In the failure cases, all three models struggle with extremely low-resolution images, severe motion blur, or handwritten text. However, SVTRv2X exhibits higher robustness, producing fewer errors while maintaining clear separability between characters. These visual comparisons indicate that the integration of the Jumble Module, Self-Distillation Module, and MoE Module in SVTRv2X enhances intra-class compactness and inter-class separability, resulting in more reliable recognition performance than existing state-of-the-art models.

Overall, the visual analysis demonstrates that SVTRv2X not only outperforms ABINet and TransOCR in recognizing visually similar or complex characters but also maintains more stable performance in diverse and challenging real-world scenarios.

### 5.2 Visualization-based analysis of similar Chinese characters

**Fig 7:**
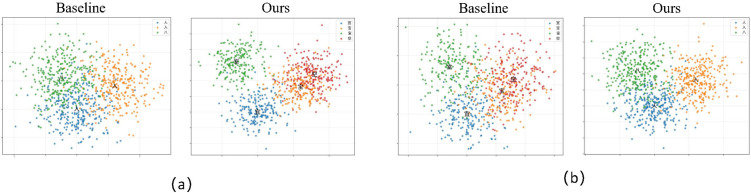
t-SNE visualization of fine-grained character clusters for baseline and SVTRv2X.

To further validate the model’s capability in fine-grained character discrimination, we conduct a dedicated analysis on two groups of visually confusing Chinese characters. The first group includes characters with highly similar bifurcated stroke structures, as shown in ‌‌the Fig 7(a). The second group includes characters that contain nested radicals and exhibit similar spatial layouts, also illustrated in the Fig 7(b). For each group, we visualize the feature embeddings generated by SVTRv2 and SVTRv2X using t-SNE.

As shown in the baseline visualizations, SVTRv2 produces scattered and overlapping clusters, indicating that similar characters are not well separated in the feature space. In contrast, SVTRv2X yields significantly more compact and clearly separated clusters, even for characters with extremely subtle structural differences. This improvement arises from our three modules: the Jumble Module enhances robustness to local stroke perturbations, the Self-Distillation Module strengthens early feature representation, and the MoE Module provides specialized processing for different character patterns.

These visual improvements align with the quantitative results, confirming that SVTRv2X substantially enhances discriminability in similar-character scenarios. The model not only reduces feature confusion within clusters but also enlarges inter-character boundaries, thereby improving recognition accuracy for fine-grained Chinese text.

### 5.3 Statistical significance analysis

To further validate the reliability and stability of SVTRv2X, we conducted multiple runs on Latin-based STR datasets, including IIIT5K, ICDAR2013, and CTR, using different random seeds. Table 5 reports the mean accuracy and standard deviation over five runs for each variant of SVTRv2X.

**Table 5:** Statistical significance testing on Latin-based STR datasets (% mean ± std over 5 runs).

Model	IIIT5K	ICDAR2013	CTR
SVTRv2X-T	99.0 ± 0.05	98.2 ± 0.07	81.64 ± 0.08
SVTRv2X-S	99.4 ± 0.04	98.8 ± 0.06	83.25 ± 0.07
SVTRv2X-B	99.6 ± 0.03	98.9 ± 0.05	84.68 ± 0.06

The results indicate that SVTRv2X achieves highly consistent performance across all datasets. The small standard deviations confirm that the observed gains are stable and reproducible, rather than being influenced by random initialization or stochastic training variations.

Specifically, SVTRv2X-T already demonstrates strong performance with mean accuracies of 99.0% on IIIT5K, 98.2% on ICDAR2013, and 81.64% on CTR. Scaling to SVTRv2X-S and SVTRv2X-B leads to further improvements, reaching 99.4% / 98.8% / 83.25% and 99.6% / 98.9% / 84.68%, respectively. These trends highlight the consistent benefit of the proposed enhancement modules—Jumble, Mixture-of-Experts, and self-distillation—in improving both accuracy and robustness across different text recognition scenarios.

This statistical significance analysis confirms that the performance improvements of SVTRv2X are not only substantial but also statistically reliable, reinforcing the robustness and generalizability of our framework on multilingual and Latin-based STR datasets.

### 5.4 Model complexity analysis

To verify the claim of efficiency preservation, we evaluated both the number of trainable parameters and the computational cost (FLOPs) of SVTRv2X variants compared to the baseline SVTRv2 models. The analysis focuses on the impact of the MoE module, which enhances model capacity while aiming to maintain efficiency.

Table 6 summarizes the comparison of model complexity between the baseline SVTRv2-B and the proposed SVTRv2X-B. SVTRv2-B has 19.2M parameters, whereas SVTRv2X-B contains 55.2M parameters. The increase is mainly due to the addition of the MoE modules, which introduce multiple expert layers and auxiliary networks to enhance feature representation. Despite this substantial increase in parameters, these modules enable richer feature extraction and model specialization, which is critical for handling diverse text patterns and challenging scenarios.

**Table 6:** Parameter and FLOPs comparison between SVTRv2 and SVTRv2X variants.

Model	Parameters (M)	FLOPs (G)
SVTRv2-B	19.2	8.22
SVTRv2X-B	55.2	8.33

In terms of computational cost, the floating-point operations only slightly increase from 8.22G for SVTRv2-B to 8.33G for SVTRv2X-B, a marginal 1.3% overhead. This indicates that although the model size is significantly larger, the MoE design effectively limits additional computation by selectively activating expert layers during forward propagation. As a result, SVTRv2X-B maintains inference efficiency comparable to SVTRv2-B while benefiting from increased capacity.

### 5.5 Design analysis of SDM

We further analyze two key design choices in the proposed SDM, including the use of 3×3 convolution and the two-stage training strategy. The 3×3 convolution is adopted to enhance local spatial interactions among neighboring patches, providing a better trade-off between representation capability and computational efficiency compared with 1×1 (no spatial modeling) and larger kernels such as 5×5 (higher cost and potential noise). For training, we employ a two-stage strategy where the model is first trained without the self-distillation loss and then fine-tuned with it. This is because early-stage features are often unstable and may introduce noisy supervision if distillation is applied too early. Delaying the distillation allows the model to learn more reliable representations, leading to improved performance and more stable convergence.

**Fig 8:**
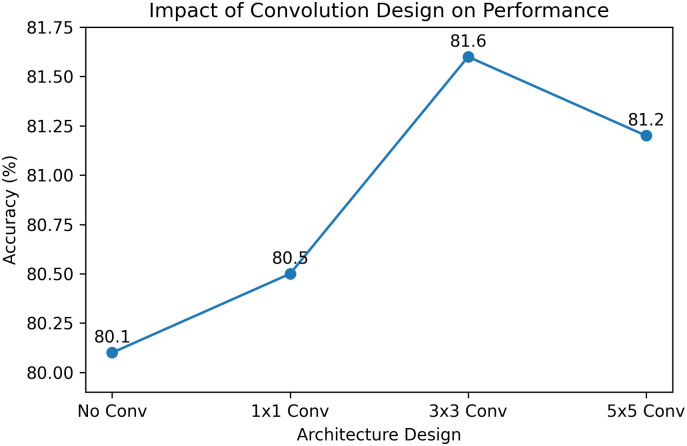
Comparison of different convolutional designs.

The effectiveness of the proposed designs can be further validated through visualization results. As shown in Fig 8, compared with alternative structures, the use of 3×3 convolution leads to higher recognition accuracy, demonstrating its superiority in modeling local spatial information and enhancing feature representation.

**Fig 9:**
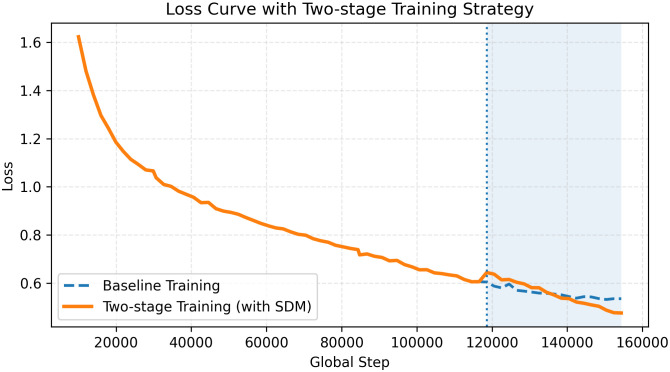
Effect of two-stage training strategy.

As illustrated in Fig 9, the training curves show that after introducing the self-distillation loss in the second stage, the overall loss is further reduced. This indicates that the proposed two-stage training strategy provides more reliable supervision and facilitates model optimization.

These results demonstrate that the proposed designs not only improve recognition performance but also lead to lower training loss and better convergence behavior during optimization.

## 6. Conclusion

In this paper, we proposed SVTRv2X, a novel and enhanced framework for scene text recognition, built upon the strong SVTRv2 baseline. Our primary contribution is the integration of three synergistic modules: the Jumble Module (JM), the Self-Distillation Module (SDM), and the Mixture of Experts (MoE) module. The Jumble Module bolsters the model’s resilience to spatial distortions, the Self-Distillation Module enriches feature representations via internal knowledge transfer, and the MoE module efficiently scales model capacity to handle diverse text patterns with a “divide-and-conquer” approach. As demonstrated through extensive experiments, SVTRv2X sets a new state-of-the-art on benchmark datasets, significantly outperforming previous methods while preserving the high inference speed characteristic of CTC-based models. Future research directions could include exploring more sophisticated MoE routing algorithms (e.g., with load balancing) and applying this modular enhancement paradigm to other visual recognition tasks. We believe SVTRv2X provides a powerful and effective blueprint for building high-performance yet efficient recognition models, and we hope our work will inspire further research in the scene text recognition field.

## Supporting information

S1 File Original Code. (ZIP)

S1 Appendix (DOCX)
