# Supplementary material for: SVTRv2X: Enhanced scene text recognition via self-distilled mixture-of-experts
Source: PLoS One. 2026 Jun 1;21(6):e0349085. doi: 10.1371/journal.pone.0349085 (PMC13225397; doi:10.1371/journal.pone.0349085)
Supplement: S1 Appendix — (DOCX) [file pone.0349085.s002.docx]

Appendix

To improve readability, the notations used in this paper are systematically summarized in Table 7. Each symbol is accompanied by a detailed explanation of its definition and usage within the proposed framework.

Table 7. Notation and Symbols

| **Symbol** | **Description** |
| --- | --- |
| *I* | Input image |
| *H*, *W*, *C* | Height, width, and channel number of *I* |
| <<Eqn61>> | Input feature sequence |
| *Y* | Target character label sequence |
| <<Eqn62>> | Valid alignment path in CTC |
| <<Eqn63>> | Set of all paths collapsing to *Y* in CTC |
| *L*_ctc_ | Connectionist Temporal Classification loss |
| *L*_sgm_ | Semantic guidance module loss |
| <<Eqn64>> | Left-to-right and right-to-left predictions at position *i* |
| <<Eqn65>> | Ground-truth character at position *i* |
| <<Eqn66>> | Intermediate feature from stage *k* |
| <<Eqn67>> | Transformed feature from stage *k* used in self-distillation |
| *L*_sd_ | Self-distillation loss |
| <<Eqn68>> | Stop-gradient operation |
| *N* | Number of supervised feature levels in self-distillation (here *N*=2) |
| <<Eqn69>> | Fraction of tokens assigned to expert *i* in MoE |
| <<Eqn70>> | Average gate probability for expert *i* |
| *L*_load_ | Load-balancing auxiliary loss for MoE |
| <<Eqn71>> | Weighting factors for total training loss components |
